# Membrane Guanylate Cyclase catalytic Subdomain: Structure and Linkage with Calcium Sensors and Bicarbonate

**DOI:** 10.3389/fnmol.2017.00173

**Published:** 2017-06-07

**Authors:** Sarangan Ravichandran, Teresa Duda, Alexandre Pertzev, Rameshwar K. Sharma

**Affiliations:** ^1^Advanced Biomedical Computing Center, Frederick National Laboratory for Cancer Research, Leidos Biomedical Research Inc., FredrickMD, United States; ^2^The Unit of Regulatory and Molecular Biology, Research Divisions of Biochemistry and Molecular Biology, Salus University, Elkins ParkPA, United States

**Keywords:** membrane guanylate cyclase, phototransduction, cyclic GMP, second messenger, signal transduction

## Abstract

Membrane guanylate cyclase (MGC) is a ubiquitous multi-switching cyclic GMP generating signaling machine linked with countless physiological processes. In mammals it is encoded by seven distinct homologous genes. It is a single transmembrane spanning multi-modular protein; composed of integrated blocks and existing in homo-dimeric form. Its core catalytic domain (CCD) module is a common transduction center where all incoming signals are translated into the production of cyclic GMP, a cellular signal second messenger. Crystal structure of the MGC’s CCD does not exist and its precise identity is ill-defined. Here, we define it at a sub-molecular level for the phototransduction-linked MGC, the rod outer segment guanylate cyclase type 1, ROS-GC1. (1) The CCD is a conserved 145-residue structural unit, represented by the segment V^820^-P^964^. (2) It exists as a homo-dimer and contains seven conserved catalytic elements (CEs) wedged into seven conserved motifs. (3) It also contains a conserved 21-residue neurocalcin δ-modulated structural domain, V^836^-L^857^. (4) Site-directed mutagenesis documents that each of the seven CEs governs the cyclase’s catalytic activity. (5) In contrast to the soluble and the bacterium MGC which use Mn^2+^-GTP substrate for catalysis, MGC CCD uses the natural Mg^2+^-GTP substrate. (6) Strikingly, the MGC CCD requires anchoring by the Transmembrane Domain (TMD) to exhibit its major (∼92%) catalytic activity; in isolated form the activity is only marginal. This feature is not linked with any unique sequence of the TMD; there is minimal conservation in TMD. Finally, (7) the seven CEs control each of four phototransduction pathways- -two Ca^2+^-sensor GCAPs-, one Ca^2+^-sensor, S100B-, and one bicarbonate-modulated. The findings disclose that the CCD of ROS-GC1 has built-in regulatory elements that control its signal translational activity. Due to conservation of these regulatory elements, it is proposed that these elements also control the physiological activity of other members of MGC family.

## Introduction

At the time of discovery (Paul et al., 1987) and molecular cloning of the first membrane guanylate cyclase (MGC), ANF-RGC’s (Chinkers et al., 1989; Lowe et al., 1989; Duda et al., 1991; reviewed in Sharma, 2002, 2010) hydropathic analysis predicted that the protein is composed of three general domains: Extracellular (ExtD), Transmembrane (TMD), and Intracellular (ICD). ICD was further subdivided into two vaguely defined domains, N-terminal KHD (Kinase Homology Domain) and C-terminal catalytic domain. Progression in the field refined this overly simplistic demarcation of these two ICD sub-domains (reviewed in Sharma and Duda, 2014) and demonstrated that KHD terminology is imprecise because it refers to a broad and complex regulatory structure. Notably, at the time it was named so because it showed significant sequence identity with the family of tyrosine protein kinases. It was later realized that the original KHD region contained at its C-terminus a 43-residue α-helical region that does not bear any sequence identity with the tyrosine kinases, hence it was not part of the KHD but independent, distinct domain of the guanylate cyclase.

It was proposed that this inter-domain region, wedged between the KHD and the catalytic domain, constitutes the dimerization domain (DD) (Garbers, 1992). It is conserved among the MGC family and functionally causes dimerization of the catalytic domain transforming it into catalytically active form (Wilson and Chinkers, 1995). This concept was then broadened and applied to define the mechanism by which it regulates the activity of ROS-GC1 (Ramamurthy et al., 2001). The central theme of this concept was that the native isolated catalytic domain exists in its inactive form and DD transforms it into an active dimeric form.

**FIGURE 1:**
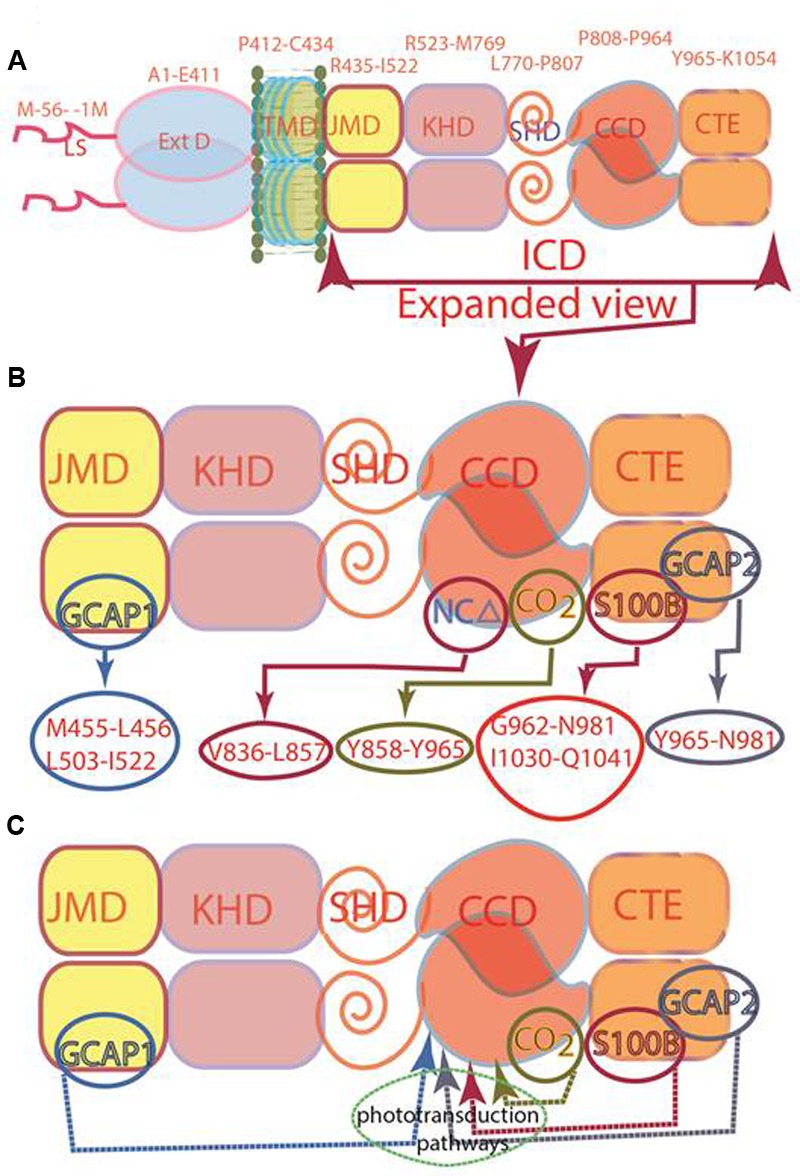
Modular composition of the ROS-GC1 dimer. **(A)** A 56 amino acid leader sequence (LS) precedes the extracellular domain (ExtD) in the nascent, immature protein. All signaling events occur in Intracellular Domain (ICD), which is separated from ExtD by transmembrane domain (TMD). ICD is composed of the following domains: juxtamembrane (JMD), kinase homology (KHD), signaling helix (SHD), catalytic core (CCD) and C-terminal extension (CTE). **(B)** ICD view expanded. Two specific switches for Ca^2+^ sensing subunits, one for GCAP1 in the JMD, and one for GCAP2 in the CTE, are located on opposing sides of the CCD. The S100B sensing site partially overlaps with that for GCAP2. Neurocalcin δ (NCδ) and CO_2_/bicarbonate recognition sites are within the CCD. The MGC complex exists as a homodimer. **(C)** Phototransduction-linked pathways. ROS-GC1 is linked with phototransduction through four distinct pathways: two modulated by Ca^2+^-sensors, GCAP1 and GCAP2; one by Ca^2+^-sensor, S100B and, the fourth, by Ca^2+^-independent CO_2_/bicarbonate. The migratory patterns of these pathways from their sites of origin to the CCD are indicated by arrows.

Later studies demonstrated that this concept is not valid. The isolated form of the recombinant ROS-GC’s catalytic domain, G^817^-Y^965^, without so called DD, is intrinsically homodimeric and is biologically active (Venkataraman et al., 2008), similar is the case with the catalytic domain of STa-RGC (Saha et al., 2009). The current consensus is that the initially named DD is a conserved five-heptad linker region which is universally present between two signaling domains, and accordingly, it has been termed signaling helix domain (SHD) (Anantharaman et al., 2006). The conclusion is that SHD does not govern the dimerization state of the MGCs, and the catalytic domain devoid of this domain exists as a dimer in its natural and crystal states (Rauch et al., 2008; Venkataraman et al., 2008; Winger et al., 2008; Saha et al., 2009). Thus, SHD is not a signature DD and has no role in the basal catalytic activity of a guanylate cyclase. Consequently, a correction has been made in our earlier illustration (Figure 4 of Sharma et al., 2016). In the newly presented illustration SHD has been depicted in its monomer form (**Figure 1**).

### Core Catalytic domain (CCD)

Originally, in ANF-RGC, the entire stretch beyond the “KHD” was termed as CCD (Chang et al., 1989; Chinkers and Garbers, 1989; Chinkers et al., 1989; Lowe et al., 1989; Duda et al., 1991). However, discovery of the ROS-GC subfamily changed this picture. There, CCD was followed by an extra C-Terminal Extension (CTE) sequence (Goraczniak et al., 1994) where the targeted sites of its two Ca^2+^ sensors, GCAP2 and S100B, were present (Duda et al., 2002, 2005). Thus, in the structural paradigm shift the CCD of ROS-GC is sandwiched between the N-terminal SHD and C-terminal CTE domains (**Figure 1C**); in this environment, CCD is able to transduce three types of phototransduction linked [Ca^2+^] signals: one, generated upstream by GCAP1, second and third generated downstream by GCAP2 and S100B (**Figure 1**; Duda et al., 2016). With an additional clue that within the CCD resides the targeted site of another Ca^2+^ sensor, neurocalcin δ (NCδ) (Kumar et al., 1999; Venkataraman et al., 2008) (**Figure 1B**), the signal transduction role of CCD expanded. CCD was no longer merely a translational center of all effector signals, it was also the regulatory element, meaning, it is a complex Ca^2+^-signaling translating element.

Guided by these cues, the boundary and the newer functional roles of CCD were reexamined. The recombinant ROS-GC1 fragment, M^733^-K^1054^ was chosen for detailed analysis (Venkataraman et al., 2008). The key findings were that: (i) CCD represents the G^817^-Y^965^ segment of the guanylate cyclase; (ii) the segment is homo-dimeric in nature; and (iii) it contains an ^844^MSEPIE^849^ NCδ regulatory motif. Incorporation of these principles in the fold recognition model of the dimeric form of CCD disclosed that CCD consists of eight β strands and six α-helices (Venkataraman et al., 2008). Its prominent feature is that the two CCD chains are antiparallel, a feature later confirmed experimentally (Duda et al., 2012b). In general, it supported its previously proposed homology-based three-dimensional CCD 1AWL model (Liu et al., 1997), yet it incorporated a significant advancement. An additional seven-residue ^911^TFRMRHM^917^ helix motif in CCD structure was present which represented the docking site, ^836^V-L^857^ (previously mis-numbered as ^837^V-L^858^) between ROS-GC1 and NCδ (Venkataraman et al., 2008).

As of to-date, no crystal structure of any MGCs CCD module exists. However, subsequent to (Venkataraman et al., 2008) publication, crystal structures of CCD of the two forms of guanylate cyclases have been solved, eukaryotic (*Cyg12*) unicellular green algae *Chlamydomonas reinhardtii* (Winger et al., 2008) and Cya2 cyanobacterium *Synechocystis* (Rauch et al., 2008). *Cyg12* represents atypical soluble and Cya2 the bacterium MGC.

With the model system of the recombinant ROS-GC1 the present study decodes the precise structure of its CCD, elucidates its biochemical principles at the sub-molecular level, through experimentation validates them for its regulation by Ca^2+^ sensors GCAP1, GCAP2 and S100B and bicarbonate operative in phototransduction, and finally, proposes their application to the general MGC family.

## Materials and Methods

### Molecular Modeling

Three-dimensional model of ROS-GC1 CCD monomer was built using structural information on eukaryotic soluble guanylate cyclase (*Cyg12*) CCD of the green algae *Chlamydomonas reinhardtii* (Winger et al., 2008) as a template, UniProt entry P55203 with A1013R as the query sequence, and Iterative Threading and ASSEmbly Refinement, I-TASSER (web server version)^1^. The top-3 unique templates identified by I-TASSER were (PDB IDs), 3uvj_A, 3et6_A and 4p2f_A. Note that 3uvj_A denotes PDBID_ChainName. Based on the secondary structure predictions and I-TASSER C-score, the top-ranked model for CCD was chosen as a representative structure referred to it as the default model for CCD. Two copies of I-TASSER built CCD monomer models were structurally aligned with the experimental soluble guanylate cyclase dimer structure, PDI ID: 3et6 (Winger et al., 2008) to create a homo-dimer ROS-GC1-CCD model. FATCAT^2^ method was used for the structural alignments and creating the dimer model of CCD. The details of the modeling are provided in the Supplemental Materials.

### ROS-GC1 Mutants

**(1) Point mutations** for the creation of the D^834^A, E^874^A, D^878^A, R^925^A, C^946^A, N^953^A, R^957^A, and E^874^A/C^946^A mutants were introduced to ROS-GC1 cDNA by polymerase chain reaction using appropriate mutagenic primers. The mutations were verified by sequencing. **(2) Membraneous abridged forms of ROS-GC1**: Δ*ExtD* mutant was constructed by introducing two *Hpa*1 restriction sites at nucleotide positions 241 and 1446 in ROS-GC1 cDNA. The 1.2 kb *Hpa*1 fragment was excised, and the remaining part re-ligated; Δ*ExtD*,Δ*JMD*,Δ*KHD* mutant was constructed from the ΔExtD mutant by introducing two *Bgl*II restriction sites at nucleotide positions 1557 and 2503 and excision of a 1 kb *Bgl*II fragment (amino acids 447–761); for the Δ*SHD* mutant two *Hpa*I sites were introduced at nucleotide positions 2533 and 2663 allowing excision of a fragment amino acid residues 772–808; for the Δ*CTE* mutant a TGA STOP codon was introduced at position 972. **(3) Soluble constructs of ROS-GC1**: *partKHD/SHD/CCD/CTE* (aa 733–1054) fragment was amplified by PCR from the ROS-GC1 cDNA by PCR and cloned in frame into pET30a bacterial expression vector; *CCD/CTE* (aa 817–1054), *CCD* (aa fragment 817–965), and *CTE* (aa fragment 986–1054) fragments were amplified by PCR from ROS-GC1 cDNA and cloned in frame into pET30aLIC vector.

### Expression of Membraneous ROS-GC1 Mutants in COS Cells

COS-7 cells were induced to express ROS-GC1 or its membrane-bound mutants using a calcium-phosphate coprecipitation technique (Sambrook et al., 1989). Sixty hours after transfection, the cells were harvested and their membranes prepared (Duda et al., 2016). The mutations did not affect membrane targeting of the proteins and their half-lives as verified by immunostaining. Some of the harvested cells were seeded on coverslips, fixed in 4% paraformaldehyde, stained with ROS-GC1 antibody and the immunoreaction was visualized after incubation with secondary antibody conjugated with DyLight488. The membraneous expression of the mutants was comparable.

### Expression of Soluble ROS-GC1 Constructs

The soluble ROS-GC1 constructs were individually expressed in BL21 bacterial cells as a His-tag fusion proteins and purified by Ni affinity chromatography. Purity of the protein was analyzed by SDS-PAGE. Concentration of the protein was determined by Bradford method at A_600_.

### Assay of Guanylate Cyclase Activity

Membrane samples were incubated individually without or with varying concentrations of recombinant bovine GCAP1 or GCAP2 (purified as described in Duda et al., 2011), recombinant mouse S100B (purified as in Pozdnyakov et al., 1997) or NaHCO_3_. The assay mixture (25 μl) consisted of (mM): 10 theophylline, 15 phosphocreatine, and 50 Tris-HCl; pH 7.5, and 20 μg creatine kinase (Sigma). In experiments with GCAP1 and GCAP2, 1 mM EGTA was added to the reaction mixture; with S100B, 1 μM Ca^2+^ was present, and when the bicarbonate effect was tested, neither EGTA nor Ca^2+^ were added. The reaction was initiated by addition of the substrate solution (4 mM MgCl_2_ and 1 mM GTP, final concentrations) and maintained by incubation at 37°C for 10 min. The reaction was terminated by the addition of 225 μl of 50 mM sodium acetate buffer, pH 6.2, followed by heating on a boiling water bath for 3 min. The amount of cyclic GMP formed was determined by radioimmunoassay (Nambi et al., 1982). All assays were done in triplicate and except where stated otherwise, were performed three times.

The catalytic activities of the soluble ROS-GC1 deletion mutants were assayed identically, except that purified protein instead of cell membranes was present in the reaction mixture and the cyclic GMP formed was measured by radioimmunoassay.

The guanylate cyclase activity is presented as average ± SD of three experiments done in triplicate.

To correlate the catalytic changes brought about by the mutations, the activities of the mutants were compared with wild type recombinant ROS-GC1 through Michaelis plots for the ligand used, fitting the data to the Hill equation, *v* = *V*_max_ (*S*)*^n^*/*K*_M_ + *S^n^*. *V*_max_ is the activity, *S* is the concentration of the ligand, *K*_M_ is the substrate concentration at which half-maximal velocity is achieved, and *n* is the Hill coefficient.

## Results

### Membrane Guanylate Cyclase ROS-GC1 Core Catalytic Domain Structure

#### ROS-GC1 CCD Is a 145-Residue, V^820^-P^964^, Structural Unit

Through the years since its discovery, the boundaries of the ROS-GC1 CCD have been progressively narrowed down. The first description states that “residues 759 to 1010, cover a region with high degree of sequence identity with the conserved catalytic regions of other guanylate and adenylate cyclases” (Goraczniak et al., 1994). It was later determined that the region beyond Y^965^ of ROS-GC1 does not contribute to the cyclase catalytic activity (Duda et al., 2002), thus this residue marks the CCD C-terminal boundary. The N-terminus of the CCD was determined through activity analyses of abridged forms of ROS-GC1. It was found to be G^817^ (Venkataraman et al., 2008).

**FIGURE 2:**
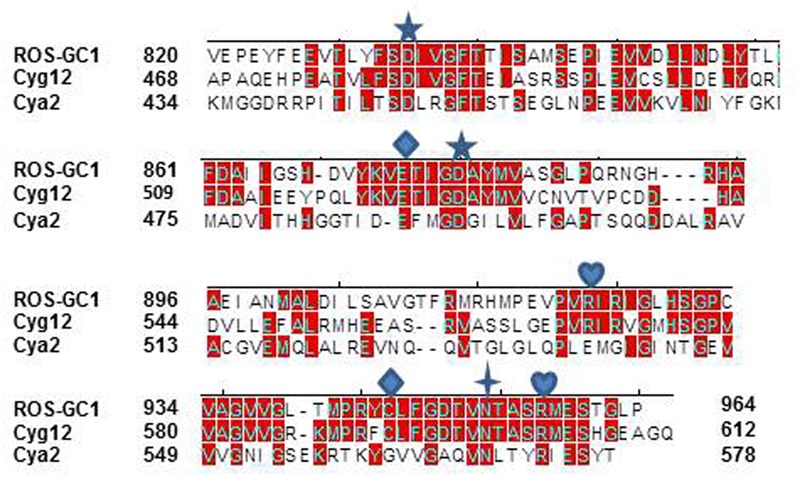
Sequence alignment of ROS-GC1 145 amino acid residues CCD with the corresponding domains of eukaryotic green algae *Chlamydomonas reinhardtii* (*Cyg12*) atypical soluble guanylate cyclase and cyanobacterium *Synechocystis* (Cya2) membrane guanylate cyclase (MGC). The sequences were aligned using Clustal V method. The conserved amino acid residues are marked in red. The numbering of ROS-G1 residues corresponds to the mature protein; of *Cyg12* and Cya2 is according to GenBank accession numbers XP_001700847 and Swiss-Prot entry P72951, respectively. The seven critical for catalytic activity residues are marked as: 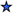 predicted to be involved in Mg^2+^ binding; 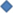 predicted to be involved in guanine recognition; 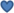 predicted to be involved in ribose binding; 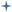 predicted to be involved in triphosphate binding.

To verify the precision of setting the ROS-GC1 CCD boundaries to G^817^ and Y^965^ as its N- and C-termini, its sequence was aligned with the corresponding CCDs of *Cyg12* (atypical soluble) and Cya2 (transmembrane), the first crystalized guanylate cyclase catalytic domains. The alignment demonstrates that the maximally conserved region stretches from ROS-GC1 amino acid residue V^820^ to P^964^ (**Figure 2**). This 145-residue region shows 45% identity with the atypical green algae soluble and 12% with the bacterium MGC CCD. The identities between *Cyg12* and Cya2 CCDs are 19%. Thus, on the evolutionary ladder the atypical soluble green algae CCD is closer to the mammalian (ROS-GC) CCD than is the bacterial MGC CCD.

#### Structure-Focused View of the CCD. Inactive and Active States

Because of the higher sequence identity, crystal structure of *Cyg12* CCD was chosen as a template to build *ab initio* a three-dimensional model of ROS-GC1 CCD. The structure of the CCD monomer was modeled by sequential substitution of the *Cyg12* residues with those of ROS-GC1 using the I-TASSER modeling suite.

**FIGURE 3:**
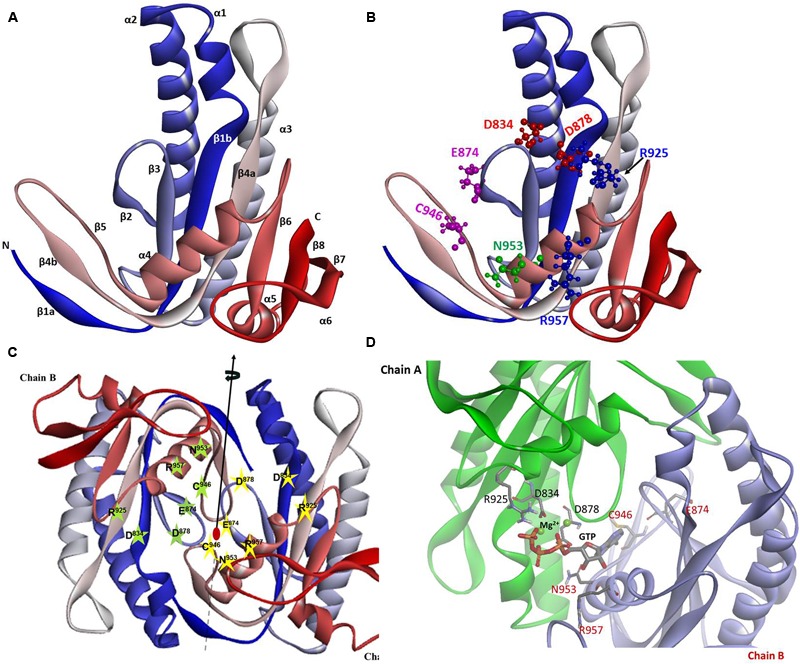
ROS-GC1 CCD 3-dimensional model. **(A)** Monomer model. The model was built *de novo* using *Cyg12* catalytic domain as a template and I-TASSER server. The model is shown in solid ribbon display style and colored using N (blue)-to-C (red) scheme. The secondary structures, eight β-stranded sheets and six α-helices are labeled. Numbering scheme of the β-strands and α-helices is similar to Winger et al. (2008). **(B)** Localization of CE residues within the CCD monomer. CCD is shown in ribbon style colored in N (blue)-to-C (red) gradient color. The amino acid residues that constitute the catalytic elements (CEs) are identified in the ball-and-stick representation. The Mg^2+^ ion binding residues (D^834^, D^878^) are shown in red, the ribose binding residue (N^953^) is shown in green, the two guanine binding residues (D^874^, C^946^) are shown in purple and the two triphosphate binding residues (R^925^, R^957^) are shown in blue. The residue locations follow UniProt P55203 canonical sequence. **(C)** CCD dimer. To create the ROS-GC1 CCD homodimer, two copies of the monomer models were independently aligned to separate chains of the *Cyg12* catalytic domain dimer (PDB ID 3et6). Protein monomer chains are shown in solid ribbon mode and colored using N-to-C coloring scheme (N-terminus: blue; C-terminus: red; a gradient color from blue through white to red for the intermediate residues). Locations of the CE residues within the dimer are indicated. The dimer possesses two-fold symmetry with a rotational axis that is perpendicular to the plane of the figure. The symmetry is represented by a filled-oval shape placed at the origin and an arrow passing through the plane. **(D)** Close-up view of the CCD active site. Model of one active site of ROS-GC1 CCD dimer with CE residues shown in a stick mode is depicted. To identify the potential binding site, the ROS-GC1 CCD model was aligned with the experimental mammalian adenylate cyclase structure (PDB: 1CJU).

The constructed model shows that the CCD monomer of ROS-GC1 retains the structural features of the class III nucleotide cyclase fold. It covers a 8-stranded β-sheet enclosed by six α-helices (**Figure 3A**). The monomer contains seven, predicted to be critical for catalytic activity (Liu et al., 1997), residues. Their positions are indicated in **Figure 3B**. They are: D^834^ in β1, D^878^ in β2 - β3 loop, N^953^ in α4, E^874^ in β2, C^946^ in β5, R^925^ and R^957^ in β4a. It is noteworthy that in the bacterial Cya2 MGC only five of them are conserved (Rauch et al., 2008) (**Figure 2**). Two residues, corresponding to ROS-GC1, R^925^ and C^946^, have been substituted by G and E residues (**Figure 2**).

To create the ROS-GC1 CCD homodimer, two copies of the monomer models were independently aligned to separate chains of the *Cyg12* catalytic domain dimer.

In the dimer form the two CCD monomer chains are locked in an antiparallel orientation and are spatially linked by two-fold symmetry axis that runs through the central dimer gap forming a circlet-like structure (**Figure 3C**). The antiparallel orientation of the two CCD monomers was experimentally documented previously (Duda et al., 2012b). The β1a, β4b, and β5 segments are part of the dimer interface. The central cavity between the two monomers includes two symmetrical active sites. Each active site is formed by critical for catalytic activity residues from both monomers (**Figure 3D**). In an inactive state the dimer is in an open conformation. It must close to attach the GTP for catalysis to occur. The two active sites in CCD are predicted to act cooperatively (Winger et al., 2008).

#### Catalytically Active Residues

Guided by the adenylate cyclase crystal structure template (Sunahara et al., 1997), it was predicted, based on human and bovine forms of ROS-GC1 CCD, that its seven residues are critical for the guanylate cyclase catalytic activity (Liu et al., 1997; Venkataraman et al., 2008). [It is, however, noted that the structure used for the modeling of the MGC (Shyjan et al., 1992) by Liu et al. (1997) is erroneous; its correct structure has been subsequently published (Goraczniak et al., 1994).] All these ROS-GC1 residues are present in the 145-residue region of ROS-GC1 CCD (**Figures 2, 3B**) and are also fully conserved in the *Cyg12* CCD (Winger et al., 2008).

It is predicted that these seven catalytic residues collectively control the basal and the ligand-dependent regulatory activities of the guanylate cyclase. In ROS-GC1 their projected functions are as follows: D^834^ and D^878^ – Mg^2+^ binding; N^953^ – ribose- positioning; E^874^ and C^946^ – guanine recognition; and R^925^ and R^957^ – triphosphate-angling, (**Figures 2, 3B**). They are termed from here on the CCD-Catalytic Element (CE) residues.

### Experimental Validation of the Model-Predicted CEs

Photoreceptor ROS-GC1 is linked with phototransduction through its four limbs by distinct pathways: two modulated by Ca^2+^-sensors, GCAP1 and GCAP2; one by Ca^2+^-sensor, S100B and, the fourth by CO_2_ (bicarbonate) via a Ca^2+^-independent mechanism (**Figure 1C**) (reviewed in Sharma et al., 2016). The first two pathways are specific for rod photoreceptors (Makino et al., 2008, 2012; Koch and Dell’orco, 2013); GCAP1- and S100B-modulated for the cone photoreceptors (Wen et al., 2012; Sharma et al., 2014) and the GCAP1 and CO_2_/bicarbonate-modulated pathways for the red cone photoreceptors (Duda et al., 2015). Notably, the migratory patterns of these pathways are very different, yet they all are translated to generate cyclic GMP at a common CCD center. The origin and flow of the GCAP1 signal pathway is exceptional, it originates in an intracellular, JMD site and is then successively processed at the KHD and SHD sites before being transmitted to the CCD for final processing operation (**Figure 1C**). In contrast, GCAP2 and S100B signals originate on the CTE and then are transmitted to the CCD. A ^657^WTAPELL^663^ motif is critical for the signaling of both GCAPs, however, it has no role in controlling the basal catalytic activity of the cyclase or in the binding of the GCAPs (Duda et al., 2011). Bicarbonate signal, in a unique mode to itself, originates and gets translated at the CCD (Duda et al., 2015, 2016). The CENTRAL POINT is that all the transmitted signals, once they arrive at the CCD, are translated by the identical transduction steps. The functional (regulatory) specificity of the signals resides only in their migratory pathways.

In order to validate the critical role of the model-predicted CE residues in ROS-GC1 function in photoreceptor outer segments, we analyzed the effect of their mutations on the basal and regulatory catalytic modes of the guanylate cyclase. Each residue was individually mutated to alanine and the resulting mutant was assessed for its basal and ligand (Ca^2+^ sensor GCAPs and S100B or Ca^2+^- independent bicarbonate)-dependent activities. Because the site-directed mutation/expression results are very similar for each CE residue, only their combined essentials are provided.

**FIGURE 4:**
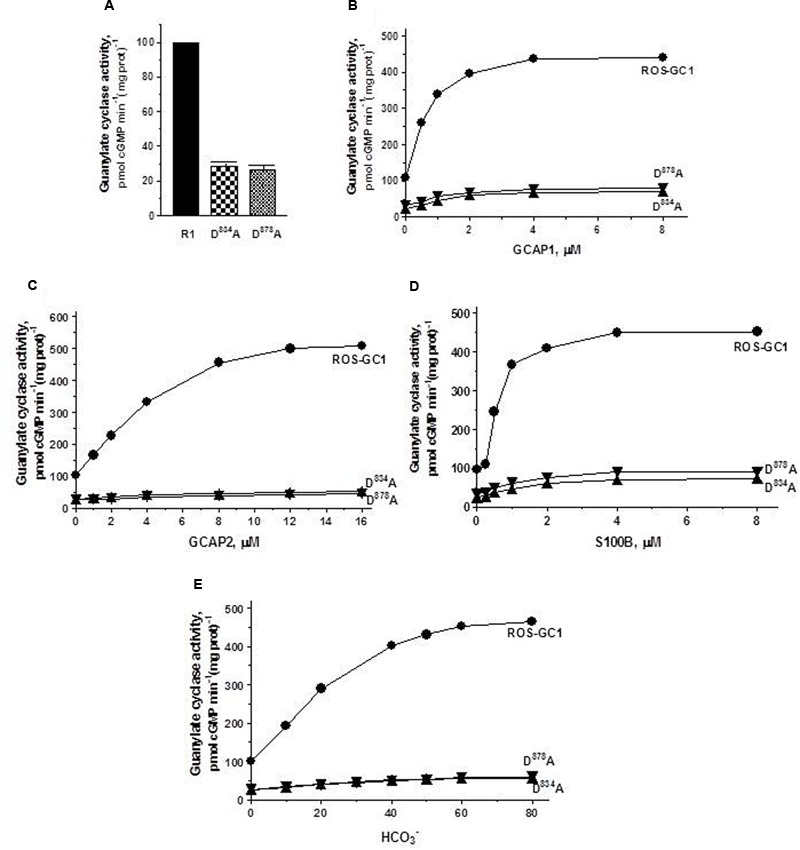
The effect of alanine mutation of Mg^2+^ coordinating residues D^834^ (CE1) and D^878^ (CE3) on basal and regulated ROS-GC1 activity. COS cells were individually transfected with ROS-GC1 mutants D^834^A or D^878^A and their membrane fractions were assessed for guanylate cyclase activity. **(A)** Basal guanylate cyclase activity; **(B)** GCAP1-dependent activity in the presence of 1 mM EGTA (10 nM Ca^2+^); **(C)** GCAP2-dependent activity in the presence of 1 mM EGTA (10 nM Ca^2+^); **(D)** S100B-dependent activity in the presence of 1 μM Ca^2+^; and **(E)** bicarbonate-dependent activity. Membranes of COS cells transfected with wild type ROS-GC1 were analyzed identically. The experiment was done in triplicate and repeated three times with different COS cell membranes preparations. The results shown are mean ± SD of these experiments. The error bars are within the size of the symbols.

*The Mg^2+^ binding residues: D^834^, CE motif 1; D^878^, CE motif 3.* D^834^ mutation results in the inhibition of 71.3% and D^878^ of 73.7% of basal ROS-GC1 catalytic activities (**Figure 4A**). Thus, each residue controls more than 70% of the basal saturation activity of ROS-GC1.

Connected with these losses, the two mutations also disable all of the ROS-GC1’s four-limbed modulatory activities. ***GCAP1***: D^834^ mutation lowers the ROS-GC1 *V*_max_ from 440 to 71 pmol cyclic GMP min^-1^ (mg protein)^-1^ and D^878^ mutation to 81 pmol cyclic GMP min^-1^ (mg protein)^-1^ (**Figure 4B**). The EC_50_ values (∼0.8 μM) and Hill coefficients (∼2) for both mutants remain unchanged. ***GCAP2***: D^834^ mutation lowers ROS-GC1 *V*_max_ from 504 to 53 pmol cyclic GMP min^-1^ (mg protein)^-1^ and D^878^ mutation, to 45 pmol cyclic GMP min^-1^ (mg protein)^-1^ (**Figure 4C**). The EC_50_ values (∼4 μM) remain the same, yet Hill coefficient values are lowered, to 1.35. ***S100B*: Recall**, In contrast to the GCAP sensors, S100B senses and stimulates ROS-GC1 catalytic activity in a Ca^2+^-dependent manner with a *K*_1/2_ of 0.3 to 0.8 μM (reviewed in Sharma et al., 2016). D^834^ mutation lowers the ROS-GC1 *V*_max_ from 452 to 72 pmol cyclic GMP min^-1^ (mg protein)^-1^ and D^878^ to 91 pmol cyclic GMP min^-1^(mg prot)^-1^ (**Figure 4D**). The S100B EC_50_ value of 0.8 μM and stimulatory Hill’s coefficient of 2 remain unchanged. ***CO_2_*:** This phototransduction-linked limb of the ROS-GC1 is a recent discovery (Duda et al., 2016; reviewed in Sharma et al., 2016). It is a bicarbonate-modulated Ca^2+^-independent signal transduction pathway. Our scattered ongoing studies have begun to show that this pathway is signaled by CO_2_ through carbonic anhydrase (CAII) enzyme, which converts CO_2_ to bicarbonate and, bicarbonate, in turn, serves the second messenger of CO_2_; importantly, in support of this hypothesis, electric recording studies demonstrate that the carbonic anhydrase inhibitor hinders the bicarbonate-dependent generated electric impulses in the red cones of the salamander (Makino et al., 2017). Like the three Ca^2+^-modulated pathways, the D^834^A and D^878^A mutations partially disable the bicarbonate modular operation (**Figure 4E**). D^834^A mutation lowers the *V*_max_ from 465 to 57 pmol cyclic GMP min^-1^(mg prot)^-1^ and D^878^A to 60 pmol cyclic GMP min^-1^(mg prot)^-1^. The EC_50_ values of ∼30 mM bicarbonate remain similar to the 25 mM for the wild type ROS-GC1. The Hill’s coefficients are 1.93 and 1.64 for the D^834^A and D^878^A mutants, respectively, slightly lower than the 2.3 for ROS-GC1.

**FIGURE 5:**
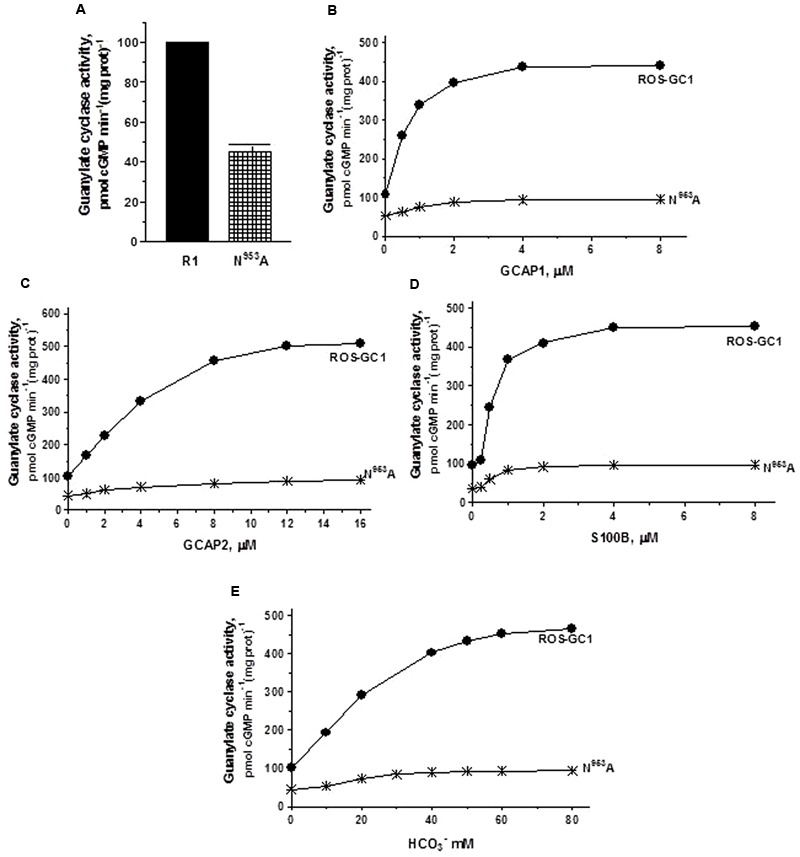
The effect of ribose binding residue N^953^ (CE6) mutation to alanine on basal and regulated ROS-GC1 activity. COS cells were transfected with ROS-GC1 mutant N^953^A and their membrane fractions were assessed for guanylate cyclase activity. **(A)** Basal guanylate cyclase activity; **(B)** GCAP1-dependent activity in the presence of 1 mM EGTA (10 nM Ca^2+^); **(C)** GCAP2-dependent activity in the presence of 1 mM EGTA (10 nM Ca^2+^); **(D)** S100B-dependent activity in the presence of 1 μM Ca^2+^; and **(E)** bicarbonate-dependent activity. Membranes of COS cells transfected with wild type ROS-GC1 were analyzed identically. The experiment was done in triplicate and repeated three times with two different COS cell membranes preparations. The results shown are mean ± SD of these experiments. The error bars are within the size of the symbols.

*The ribose positioning residue, N^953^, CE motif 6*, controls 55% of basal ROS-GC1’s catalytic activity (**Figure 5A**) and also disables most of the modulatory activities of its four-limbed pathways (**Figures 5B–E**). ***GCAP1***: N^953^ mutation lowers the ROS-GC1 *V*_max_ from 440 to 95 pmol cyclic GMP min^-1^ (mg protein)^-1^. The EC_50_ values (∼0.8 μM) and Hill coefficients (∼2) for both mutants remain the same as for wild type ROS-GC1 (**Figure 5B**). ***GCAP2***: N^953^ mutation lowers the ROS-GC1 *V*_max_ from 508 to 92 pmol cyclic GMP min^-1^ (mg protein)^-1^ (**Figure 5C**). The mutation does not affect the EC_50_ (∼4 μM) and Hill coefficient of 2. ***S100B*:** N^953^ mutation lowers the ROS-GC1 *V*_max_ from 452 to 97 pmol cyclic GMP min^-1^ (mg protein)^-1^ (**Figure 5D**). The EC_50_ value of 0.8 μM and stimulatory Hill’s coefficient of 2 remains unchanged. ***CO_2_*:** The N^953^ mutation results in lowering the ROS-GC1 *V*_max_ from 465 to 94 pmol cyclic GMP min^-1^ (mg protein)^-1^ (**Figure 5E**) but has no effect on EC_50_ (∼25 mM bicarbonate) and Hill coefficient (2.2) values.

**FIGURE 6:**
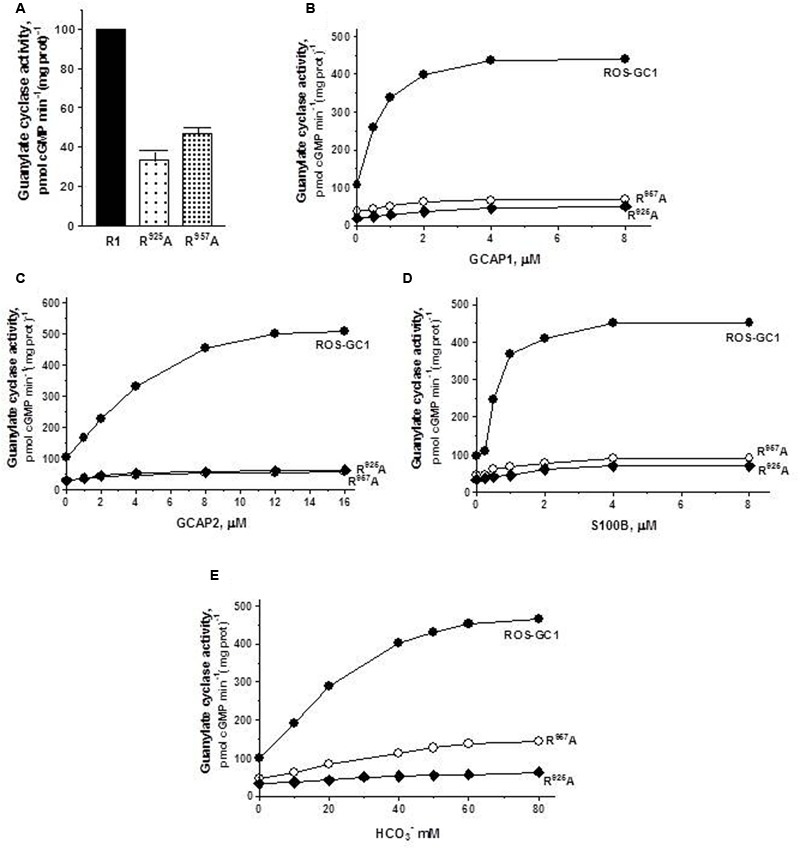
The effect of triphosphate binding residues R^925^ (CE4) and R^957^ (CE7) substitution with alanine on basal and regulated ROS-GC1 activity. Membranes of COS cells expressing separately two single mutants R^925^A or R^957^A were assayed for guanylate cyclase activity. **(A)** Basal guanylate cyclase activity; **(B)** GCAP1-dependent activity in the presence of 1 mM EGTA (10 nM Ca^2+^); **(C)** GCAP2-dependent activity in the presence of 1 mM EGTA (10 nM Ca^2+^); **(D)** S100B-dependent activity in the presence of 1 mM Ca^2+^; and **(E)** bicarbonate-dependent activity. Membranes of COS cells transfected with wild type ROS-GC1 were analyzed identically. The experiment was done in triplicate and repeated two times with different COS cell membranes preparations. The results shown are mean ± SD of these experiments. The error bars are within the size of the symbols.

*The triphosphate angling residues R^925^, motif CE4, and R^957^, motif CE7.* They control, respectively, 66 and 53% of basal ROS-GC1 catalytic activity, and also disable most of the modulatory activities of its four-limbed pathways (**Figure 6**). ***GCAP1*:** R^925^ mutation lowers the ROS-GC1 *V*_max_ from 440 to 50 pmol cyclic GMP min^-1^ (mg protein)^-1^ and R^957^ mutation to 70 pmol cyclic GMP min^-1^ (mg protein)^-1^ (**Figure 6B**). The EC_50_ values (∼0.8 μM) and Hill coefficients (∼2) for both mutants remain unchanged, however. ***GCAP2***: R^925^ mutation lowers the ROS-GC1 *V*_max_ from 508 to 63 pmol cyclic GMP min^-1^ (mg protein)^-1^ and R^957^ mutation to 57 pmol cyclic GMP min^-1^ (mg protein)^-1^ (**Figure 6C**). The EC_50_ values (∼4 μM) remain unchanged and also the Hill coefficient value of 2.0. ***S100B*:** R^925^ mutation lowers the ROS-GC1 *V*_max_ from 452 to 70 pmol cyclic GMP min^-1^ (mg protein)^-1^ and R^957^ to 90 pmol cyclic GMP min^-1^ (mg prot)^-1^ (**Figure 6D**). The S100B EC_50_ value of 0.8 μM and stimulatory Hill’s coefficient of 2 remains unchanged. ***CO_2_*:** Like the three Ca^2+^-modulated pathways, the R^925^ and R^957^ mutations disable the bicarbonate operation. R^925^ mutation lowers the ROS-GC1 *V*_max_ from 465 to 63 pmol cyclic GMP min^-1^ (mg protein)^-1^ and R^957^ to 145 pmol cyclic GMP min^-1^ (mg prot)^-1^ (**Figure 6E**). The EC_50_ for bicarbonate is ∼25 mM for both mutants. The Hill coefficient of 1.1 for the R^925^A mutant is significantly lower than 2.3 for wild type ROS-GC1 but for the R^957^A mutant, 1.7, is close.

**FIGURE 7:**
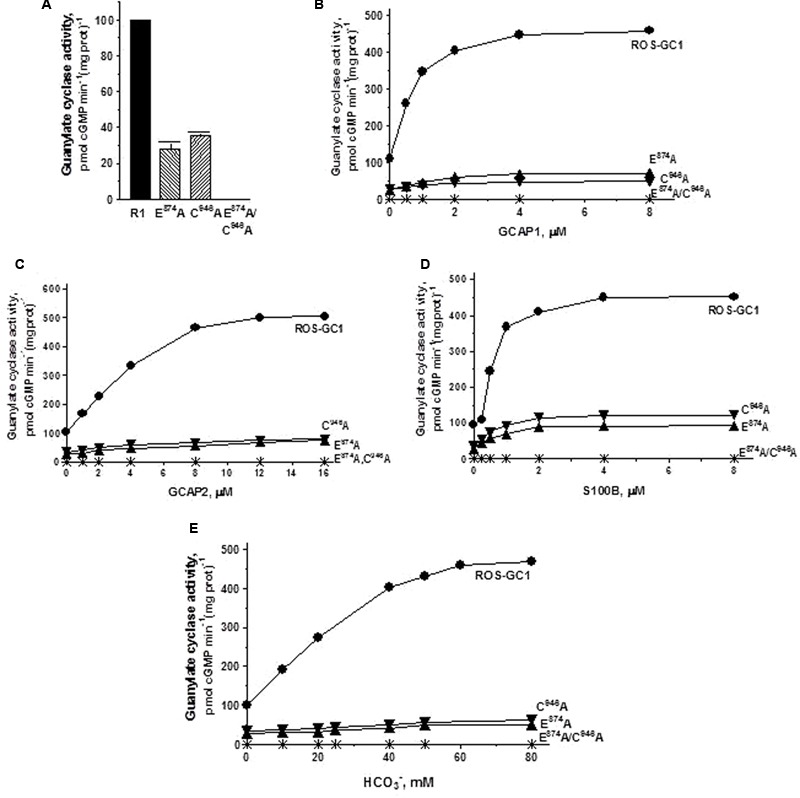
The effect of substitution of guanine binding residues E^874^ (CE2) and C^946^ (CE5) with alanine on basal and regulated ROS-GC1 activity. Membranes of COS cells induced to express independently two single mutants E^874^A or C^946^A or one double mutant E^874^A/C^946^A were assayed for guanylate cyclase activity. **(A)** Basal guanylate cyclase activity; **(B)** GCAP1-dependent activity in the presence of 1 mM EGTA (10 nM Ca^2+^); **(C)** GCAP2-dependent activity in the presence of 1 mM EGTA (10 nM Ca^2+^); **(D)** S100B-dependent activity in the presence of 1 mM Ca^2+^; and **(E)** bicarbonate-dependent activity. Membranes of COS cells transfected with wild type ROS-GC1 were analyzed identically. The experiment was done in triplicate and repeated three times with two COS cell membranes preparations. The results shown are mean ± SD of these experiments. The error bars are within the size of the symbols.

*The guanine recognition residues, E^874^, motif CE2 and C^946^, motif CE5.* Individually, residues E^874^ and C^946^ control, respectively, 72 and 65% of the basal catalytic activities (**Figure 7A**) and both mutations together disable all the basal activity of ROS-GC1 (**Figure 7A**: mutant E^874^A/C^946^A). ***GCAP1***: E^874^A mutation lowers the ROS-GC1 *V*_max_ from 440 to 115 pmol cyclic GMP min^-1^ (mg protein)^-1^ and the C^946^A mutation to 87 pmol cyclic GMP min^-1^ (mg protein)^-1^ (**Figure 7B**). The EC_50_ values of ∼0.8 μM are the same as for the wild type ROS-GC1 and the Hill’s coefficients of 1.75 and 1.71 for the mutants are close to the 2 value for the wild type cyclase. The double mutant is unresponsive to any concentration of GCAP1 tested (**Figure 7B**). ***GCAP2***: Mutation of E^874^ lowers the ROS-GC1 *V*_max_ from 508 to 60 pmol cyclic GMP min^-1^ (mg protein)^-1^ and mutation of C^946^ to 80 pmol cyclic GMP min^-1^ (mg protein)^-1^ (**Figure 7C**). The EC_50_ values (∼4 μM) remain unchanged; the Hill’s coefficients are above 1, but slightly lower than that for wild type ROS-GC1, being 1.57 for the E^874^A mutant and 1.50 for C^946^A. The double mutant does not respond to GCAP2 (**Figure 7C**). ***S100B***: E^874^A mutation lowers the ROS-GC1 *V*_max_ from 452 to 93 pmol cyclic GMP min^-1^ (mg protein)^-1^ and C^946^A, to 122 pmol cyclic GMP min^-1^ (mg prot)^-1^ (**Figure 7D**). The values of EC_50_ of ∼0.7 μM S100B and the Hill’s coefficients of 1.7 for both mutants are the same as for the wild type ROS-GC1. There is no measurable activity of the double mutant either with or without S100B (**Figure 7D**). ***CO_2_***: Like the three Ca^2+^-modulated pathways, E^874^A and C^946^A mutations affect bicarbonate operation. Mutation of E^874^ lowers the ROS-GC1 *V*_max_ from 465 to 54 pmol cyclic GMP min^-1^ (mg protein)^-1^ and C^946^ to 61 pmol cyclic GMP min^-1^(mg prot)^-1^ (**Figure 7E**). The mutations do not affect significantly the EC_50_ and Hill coefficient values. They remain comparable to the wild type ROS-GC1 values being EC_50_ ∼30 mM for both mutants and Hill coefficient 1.91 for E^874^A and 1.53 for C^946^A. Importantly, there is no detectable catalytic activity of the double mutant (**Figure 7E**).

An earlier study (Tucker et al., 1998) concluded that both residues, E^874^ and C^946^ (E^925^ and C^997^, corresponding human ROS-GC1 residues), individually control the total catalytic activity of the ROS-GC1. In this study the authors used the HEK 293 cell system for the expression of guanylate cyclases; and they did not evaluate the mutant with double mutations (E^925^/C^997^) and the residues were mutated, respectively, to K and D instead of A. Our results revise this conclusion; only two mutations together, E^874^/C^946^, totally disable the catalytic activity of ROS-GC1.

*Alanine mutation of CE residues does not affect cooperativity of CCD active sites*. Homodimeric antiparallel structure of the guanylate cyclase catalytic domain results in the existence of two equivalent catalytically active sites (Liu et al., 1997; Venkataraman et al., 2008). It was now sought to determine how mutation of an individual CE would affect the communication between the active sites in the mutant-cyclases. Activity assays were carried out in the presence of constant concentration of Mg^2+^ and varying concentrations of GTP (0–3 mM) and *K*_M_ as a measure of a mutant’s affinity for GTP and Hill coefficients indicative of interaction between active sites, were determined. The results are summarized in **Table 1**. For the wild type ROS-GC1 the *K*_M_ value for GTP was 0.45 mM. All ROS-GC1 CE mutants exhibited *K*_M_ values around 0.5 mM, ranging from 0.48 for D^878^A to 0.64 for C^946^A. As expected, ROS-GC1 exhibited positive cooperativity, with a Hill coefficient of ∼2 (2.01 ± 0.32), conforming the presence of two, interacting with each other, active sites. All the mutants exhibited also positive cooperativity, with Hill coefficients significantly above 1, varying from 1.68 ± 0.22 for C^946^A to 2.11 ± 0.23 for D^878^A. These results demonstrate that although the CE mutations considerably hinder the catalytic processes of the active sites, leading to drastically lowered *V*_max_ values, the sites remain cooperative in their catalytic functions.

**Table 1:** Effect of ROS-GC1 CE mutations on catalytic site characteristics.

	*K*_M_ (mM)	Hill’s coefficient
ROS-GC1	0.45 ± 0.02	2.01 ± 0.32
D^834^ A	0.56 ± 0.03	1.83 ± 0.37
E^874^ A	0.53 ± 0.03	2.03 ± 0.37
D^878^ A	0.48 ± 0.04	2.11 ± 0.23
R^925^ A	0.52 ± 0.03	1.98 ± 0.39
C^946^ A	0.64 ± 0.02	1.68 ± 0.22
N^953^ A	0.49 ± 0.01	1.99 ± 0.12
R^957^ A	0.5 ± 0.03	2.06 ± 0.25

*COS cells were induced to express ROS-GC1 or its mutants. Membranes of these cells were individually analyzed for guanylate cyclase activity in the presence of increasing concentrations of GTP (0–3 mM) and constant 4 mM MgCl_*2*_. The experiment was done in triplicate. *K*_*M*_ was determined graphically and Hill’s coefficients were calculated as described in “Materials and Methods” section.*

These results provide experimental proof that indeed the D^834^, E^874^, D^878^, R^925^, C^946^, N^953^, and R^957^ residues of the ROS-GC1 CCD are critical for the cyclase’s catalytic activity. Although no one mutation individually leads to complete inactivation, they collectively do so. Therefore, each of these residues represents one of the seven structural units of the CE that control full catalytic activity of ROS-GC1.

Is the same true for all MGCs?

**FIGURE 8:**
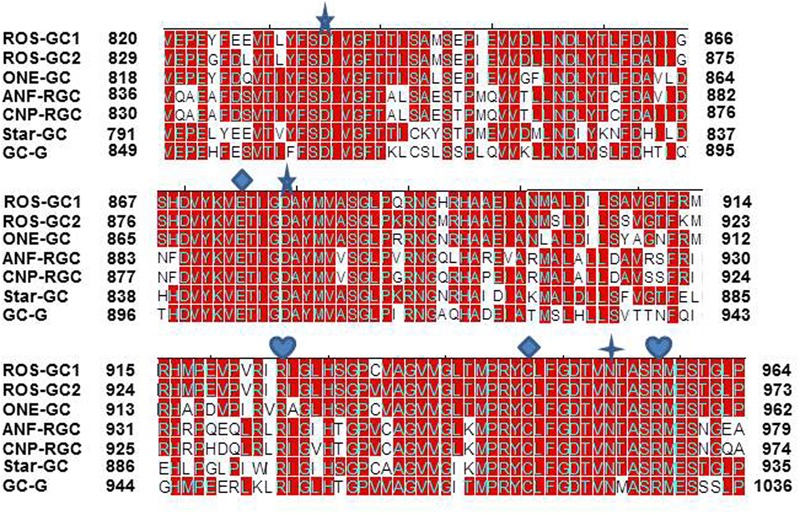
Conservation of CCD sequence among mammalian MGCs. The sequences of CCD of bovine ROS-GC1 (GC-E), bovine ROS-GC2 (GC-F), rat ONE-GC (GC-D), rat ANF-RGC (NPR-A), human CNP-RGC (NPR-B), human STa-RGC (hSTAR) and rat GC-G were aligned. The names in brackets are the alternate names according to NCBI PubMed. Positions of the amino acid residues of all these MGCs correspond to mature proteins. The conserved amino acid residues are marked in red. The seven critical for catalytic activity residues are marked as: 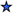 predicted to be involved in Mg^2+^ binding; 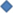 predicted to be involved in guanine recognition; 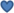 predicted to be involved in ribose binding; 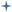 predicted to be involved in triphosphate binding.

***The CCD structure along with seven CE residues is conserved in the mammalian MGC family.*** To determine whether the CE residues are conserved in the MGC family first, the sequence of ROS-GC1 145-residue CCD was compared with the corresponding sequences of the other six members of the family: ROS-GC2, V^829-^P^975^; ONE-GC, V^818-^P^962^; ANF-RGC, Q^836-^L^980^; CNP-RGC, Q^830-^A^974^; STa-RGC, K^794-^P^935^; GC-G, V^849-^P^993^ (**Figure 8**). The percentile of their sequence identities with ROS-GC1 were: 93, 85, 68, 70, 75, and 69, respectively.

To assess and then to formulate a unified signal transduction concept by which the CEs operate, the question was asked whether in all members of the MGC family they exist in conserved structural motifs.

**FIGURE 9:**
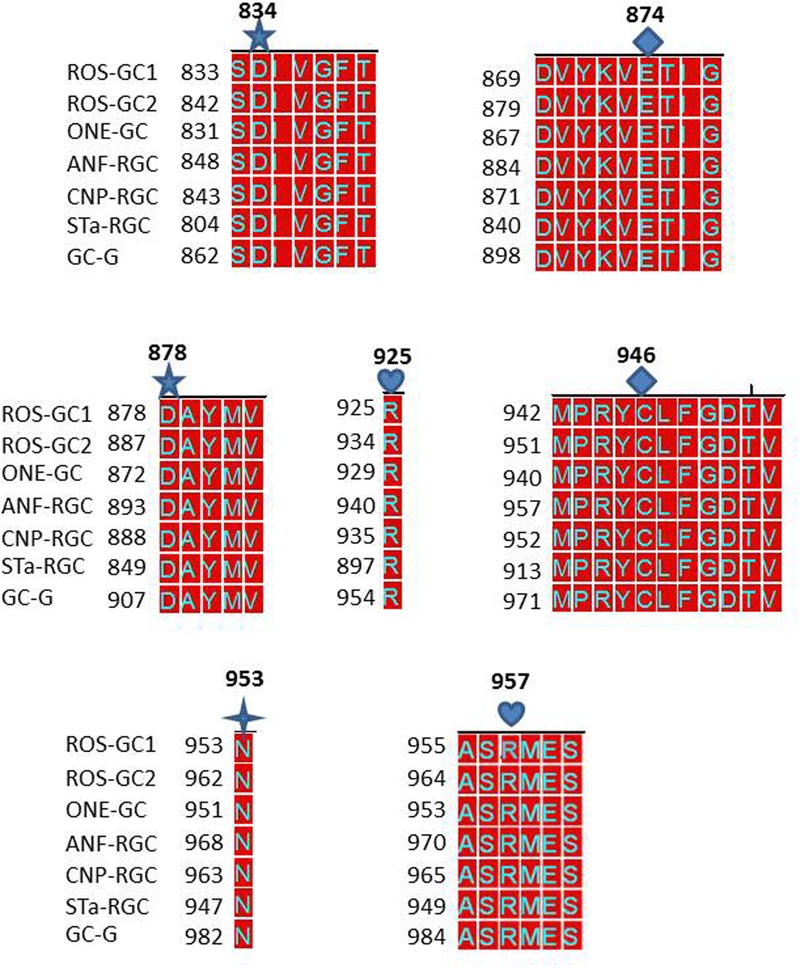
Conservation of the seven CEs of CCD in mammalian MGCs. The sequences of the CE modules of bovine ROS-GC1: CEM1, S^833^-T^839^; CEM2, D^869^-G^877^; CEM3, D^878^-V^882^; CEM4, R^925^; CEM5, M^998^-V^952^; CEM6, N^953^; CEM7, A^955^-S^960^ were aligned with the corresponding sequences of bovine ROS-GC2 (GC-F), rat ONE-GC (GC-D), rat ANF-RGC (NPR-A), human CNP-RGC (NPR-B), human STa-RGC (hSTAR) and rat GC-G. The names in brackets are the alternate names according to NCBI PubMed. Conserved residues are marked in red. The alignment demonstrates total conservation of the CEs and modules they reside in. The CEs are indicated as: 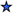 predicted to be involved in Mg^2+^ binding (CEM1 and CEM3); 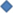 guanine recognition (CEM2 and CEM5); 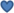 predicted to be involved in ribose binding (CEM6); 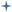 predicted to be involved in triphosphate binding (CEM4 and CEM7).

The answer was in affirmative. In ROS-GC1 the seven CEs are present in motifs: S^833^-T^839^; D^869^-G^877^; D^878^-V^882^; R^925^; M^942^-V^952^; N^953^; and A^955^-S^960^. These motifs are conserved in all seven members of the MGC family (**Figure 9**) and thus, they are termed CEs motifs, CEMs. It is logical to envision that, as in ROS-GC1, these CEMs are, respectively, involved in Mg^2+^ binding, ribose positioning, guanine recognition, and triphosphate angling. We therefore propose that for all MGCs the CEMs control their basal and ligand-dependent catalytic activities.

### CCDs of the MGC Family Embody a Conserved ROS-GC1’s 22-Residue Neurocalcin δ (NCδ)–Modulated Structural Domain

Prior to the remarkable discovery that ROS-GC1 CCD is embedded with the residence of neurocalcin δ (NCδ) recognition site (Venkataraman et al., 2008), CCD was believed to be only the translational center of the ligand-dependent signals into the production of cyclic GMP. Linkage with NCδ changed this paradigm, CCD also became a regulatory center for the NCδ-modulated Ca^2+^signals. Strikingly, in contrast to all other ligand-dependent signals, NCδ-modulated Ca^2+^ signal originates and gets transduced in CCD.

**FIGURE 10:**
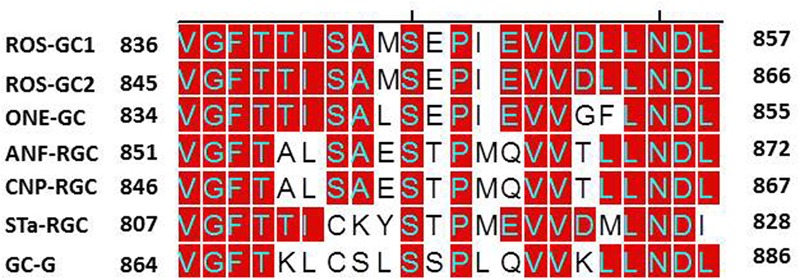
Sequence alignment of the ROS-GC1 neurocalcin δ binding site with the corresponding regions of other mammalian MGCs. Sequence of ROS-GC1 neurocalcin δ binding site, amino acid residues V^836^-L^857^, was aligned with the corresponding regions of bovine ROS-GC2 (GC-F), rat ONE-GC (GC-D), rat ANF-RGC (NPR-A), human CNP-RGC (NPR-B), human STa-RGC (hSTAR) and rat GC-G. The names in brackets are the alternate names according to NCBI PubMed. Positions of the amino acid residues of all these MGCs correspond to mature proteins. The conserved residues are marked in red.

To determine if presence of the NCδ-sensing motif, V^836^-L^857^, is a common feature of the MGC family, sequence of this motif was compared in all members of the MGC family (**Figure 10**). With 100% conservation, ROS-GC1 and ROS-GC2 preserved this motif and there was about 87% conservation in ONE-GC. With the remainder four MGCs the respective percentile conservation was: ANF-RGC 68; CNP-RGC, 68; Star-GC, 68; and GC-G, 59. In accordance with this pattern, it has been experimentally validated that like ROS-GC1, ONE-GC is Ca^2+^-modulated via its sensor myr-NCδ (Duda et al., 2001; Krishnan et al., 2004; Duda and Sharma, 2008; Sharma and Duda, 2010). Importantly, with the evidence that ANF-RGC also is Ca^2+^- modulated via its sensor myr-NCδ (Duda et al., 2012a) and its NCδ sensing motif has total sequence conservation with the corresponding region in CNP-RGC (**Figure 10**), we conclude that this motif plays an important physiological role in the regulatory property of the MGC family. Notably, total conservation of the N-terminal VGFT and C-terminal- -LND flanking regions of the NCδ sensing motif is a family trait (**Figure 10**).

### CCDs of the MGC Family House Also a Conserved ROS-GC1’s 108-Residue CO_2_-Modulated Region

Adding further complexity to the modular role of the CCD, recent studies show that ROS-GC1’s CCD also contains a 108-residue, Y^858^–Y^965^, structural element (Duda et al., 2015, 2016). Except for one residue, Y^965^, it resides within the CCD; and it represents a Ca^2+^-independent CO_2_-modulated region of the GC (**Figure 1**) (Makino et al., 2017). This region shows 70 to 95% identity in the MGC family, is also CO_2_-modulated in ONE-GC and possibly in GC-G. Thus, MGC CCD besides containing CEs, contains also two, NCδ and CO_2_, modulated regions.

### Transmembrane Domain Is a Major Contributor of the MGCs CCD Activity

Right from the time of MGC activity detection in the mammalian tissues the mystery surrounded on one of its features: why, in contrast to the adenylate cyclase which uses Mg^2+^, the preferred cofactor of MGC is Mn^2+^ (Hardman and Sutherland, 1969; Ishikawa et al., 1969; Schultz et al., 1969; White and Aurbach, 1969) (reviewed in Sharma, 2010)?

This mystery has been sustained even with the crystalized forms of the eukaryotic *Cyg12* soluble (Winger et al., 2008) and the bacterium Cya2 (Rauch et al., 2008) membrane forms. These CCDs show no significant catalytic activities when the Mg^2+^-GTP substrate is used; they only show these with the use of Mn^2+^-GTP.

**FIGURE 11:**
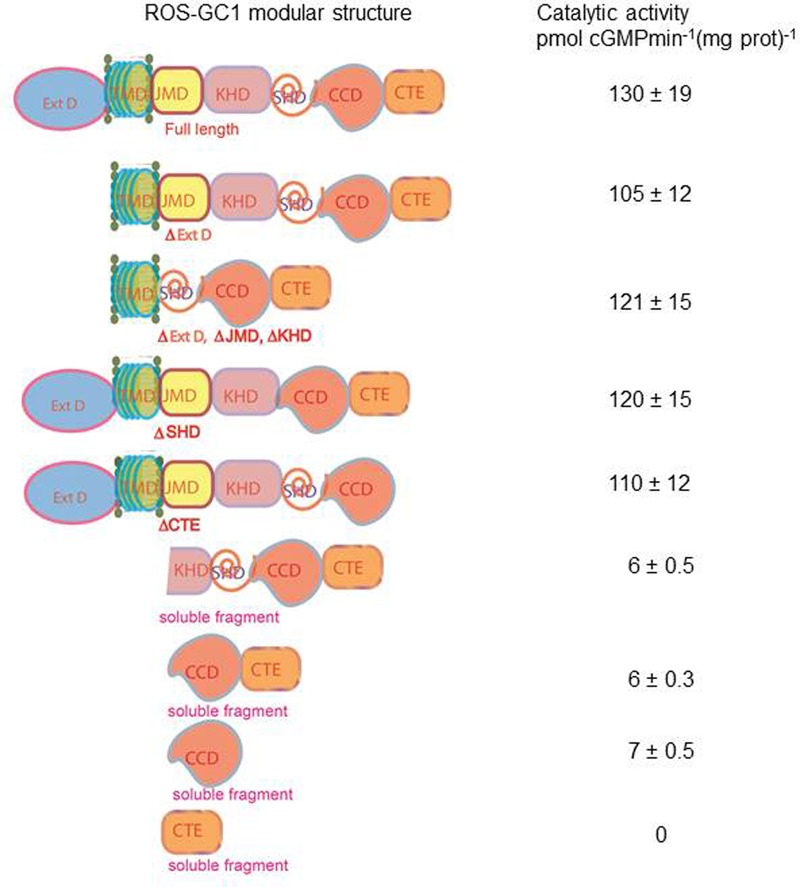
Transmembrane domain contributes to the ROS-GC1 catalytic activity. The left panel presents schematically various ROS-GC1 deletion mutants. The first five abridged forms of ROS-GC1 have retained TMD whereas the three at the bottom are devoid of TMD thus they are soluble constructs. The membraneous and soluble constructs were prepared and expressed as described in “Materials and Methods” section; the dimerization of the soluble constructs was verified by FPLC as in Venkataraman et al. (2008); for the isolated core catalytic domain mutant (CCD), the ability to form antiparallel homodimer was verified by bifunctional fluorescence (Duda et al., 2012b). They were and assayed for guanylate cyclase activity in the presence of 1 mM GTP and 4 mM Mg^2+^. The right panel provides the values of specific guanylate cyclase activity [pmol cyclic GMP min^-1^ (mg prot)^-1^] of each construct.

To address this issue, we performed domain-by-domain deletion/expression analysis of the recombinant ROS-GC1 (**Figure 11**). The catalytic activity [pmol cyclic GMP min^-1^ (mg prot)^-1^] of each truncated construct was assessed using the natural Mg^2+^-GTP as the substrate. The isolated soluble CCD construct contained only a minimal basal specific catalytic activity of 7 pmol. In contrast, the r-ROS-GC1’s basal catalytic activity and of all its truncated constructs ranged between 110 and 130 pmol, a value almost 22-fold higher than its isolated form. Importantly, whenever the constructs lacked the TMD, illustrated by the CCD–CTE and CCD, the CCD’s catalytic activity dramatically dropped from 130 to 6 pmol. To rule out the unlikely possibility that the drop in catalytic activity might be restored by anchoring CCD with any domain located N-terminally to it, the CCD’s catalytic activity was assessed in the partially truncated KHD^-^-SHD-CCD-CTE soluble fragment (**Figure 11**). No restoration occurred, however. The catalytic activity remained 6 pmol.

Our interpretation of these results is that: (1) the natural substrate of the MGC’s CCD for catalysis is Mg^2+^-GTP; (2) none of the modular domains- -ExtD, JMD, KHD, SHD and CTE- -have any role in its basic catalytic operation; and (3) TMD is the major contributor in boosting CCD’s basal catalytic activity.

**FIGURE 12:**
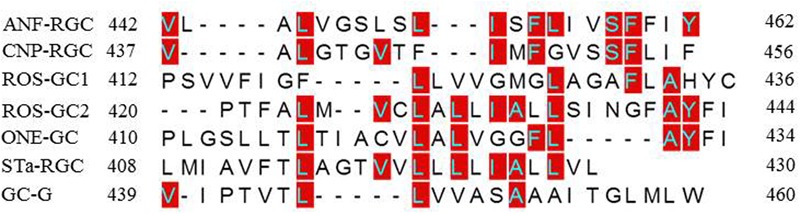
Sequence comparison of mammalian MGCs TMD. The sequences of TMDs of bovine ROS-GC1 (GC-E), bovine ROS-GC2 (GC-F), rat ONE-GC (GC-D), rat ANF-RGC (NPR-A), human CNP-RGC (NPR-B), human STa-RGC (hSTAR) and rat GC-G were aligned. The names in brackets are alternate names according to NCBI PubMed. Positions of the amino acid residues of all these MGCs correspond to mature proteins. The conserved amino acid residues are marked in red.

To understand how TMD might contribute to the CCD’s catalytic activity, the possibility was considered that this might be due to a unique consensus motif of the TMDs. Structural comparison, however, demonstrated that this was not the case (**Figure 12**). Compared to the ROS-GC1, the percentile identity between the MGCs TMDs was only marginal, ROS-GC2, 24; ONE-GC, 24; Star-GC, 22; GC-G, 32; ANF-RGC, 24; CNP-RGC, 25. It is noteworthy that not even a single residue was fully conserved among the family members. Thus, we propose that TMD contributes to the CCD’s catalytic activity by making it possible that all the successive modular domains- -JMD, KHD, SHD, and CCD- are in properly fixed positions (Top Panel: **Figure 1**), resulting in the optimal conditions for catalysis to occur.

## Discussion

Using the model system of photoreceptor ROS-GC1 the presented study (a) decodes the basic structure and biochemistry of the core unit of CCD; (b) models its three-dimensional configuration; (c) develops a general MGC family signal transduction theme; and (d) experimentally validates the theme for ROS-GC1.

### Basic Structure

Encoded by seven genes, MGC is the generator of cyclic GMP which serves as an intracellular second messenger for the countless physiological processes. It is a single transmembrane spanning protein existing as a homodimer upheld by two contact regions between the monomers (**Figure 1**). Based on the crystal structure of the ANF-RGC ExtD (Ogawa et al., 2004), the first prototype guanylate cyclase member (reviewed in Sharma et al., 2016), there is head-to-head contact of these two domains. The second contact is formed at the CCD (Venkataraman et al., 2008) where two monomers assume an antiparallel conformation (Duda et al., 2012b) and (**Figure 1**). The encoded seven MGCs- -ANF-RGC, CNP-RGC, STa-RGC, ROS-GC1, ROS-GC2, ONE-GC and GC-G- -initiate cellular signaling process from different sites, the first three from the ExtD, next two from the ICD, ONE-GC from both ExtD and ICD and GC-GC, most probably from ICD (Sharma et al., 2016). Yet, regardless of the origin, all signals are transduced and translated into the generation of cyclic GMP at the CCD (Sharma et al., 2016).

### Three-Dimensional Model

In the photoreceptor ROS-GC1, a mammalian MGC, CCD is a 145-residue structural unit stretching from V^820^ to P^964^ (**Figure 1**). In its natural form, it is homodimeric. In its simulated 3D-model, each monomer is composed of 8-stranded β-sheets and six α-helices (**Figure 3A**). In its homodimer form two monomers assume a two-fold symmetry axis with a central gap of a circlet-like shape (**Figure 3B**). The circlet between the monomers contains two symmetric active sites, each formed by the conserved residues (CEs) from the two monomers (**Figure 3C**). The CEs are: two Mg^2+^-binding, D^834^ and D^878^; one, ribose positioning, N^953^; two guanine recognition, E^874^ and C^946^; and two, triphosphate angling, R^925^ and R^957^. The projection is that in an inactive state the dimer is in an open conformation but in closed upon binding GTP for catalysis (**Figure 3D**).

### General Signal Transduction Theme

The CCD structure is conserved in all seven mammalian MGCs, showing a sequence identity between 92 and 65% (**Figure 8**). Characteristically, it is closer between the Ca^2+^-modulated guanylate cyclases – -compare ROS-GC1 vs ROS-GC2, 93% and ROS-GC1 vs ONE-GC, 85%. It is predicted that this closeness in identity is reflective of the regulatory feature of the CCD, in contrast to its basic catalytic mode, explained below.

In a uniform general mode, all seven CEs of all MGCs are arranged in structural motifs (**Figure 9**). These motifs, respectively, represent the common features of all MGCs. This structured theme of the motifs points out that they in an identical fashion control the folding patterns of all MGCs and, thus, their basic guanylate cyclase catalytic activities.

The presented study also discloses two important features of the mammalian MGC CCD.

(1) An extraordinary characteristic that sets the mammalian MGC family apart from the soluble (Winger et al., 2008) and the bacterium MGC (Rauch et al., 2008) is that the mammalian CCD uses natural Mg^2+^-GTP as a substrate for catalysis. It is understandable for the bacterium MGC’s CCD to use Mn^2+^-GTP because bacteria need this trace element for survival. Yet, it is surprising in the case of eukaryotic soluble guanylate cyclase, *Cyg12*, because this cation is not its natural substrate for catalysis.
(2) To this moment, it has been a mystery as to why the basal catalytic activity of the MGC CCD drops about 90% in its’ isolated from. In a major contribution, this study solves this riddle. The solution resides in its TMD. The TMD anchors it, fixes its conformation and makes it more amenable to the signal transduction events involved in controlling its basal and catalytic events. Importantly, the possibility that this characteristic might be due to a unique signaling characteristic of a structural element in the TMD has been ruled out because such an element does not exist in TMD (**Figure 12**).

### Predictions, Experimentally Validated

In accordance with the predictions, (i) MGC CCD is homo-dimeric. (ii) Seven CEs embedded in their seven CE motifs, all are critical in controlling the basal catalytic activity of the MGCs. (iii) Mg^2+^-GTP is the natural substrate for catalysis, this basic operation is controlled by the CEM motifs, 2 and 3. (iv) The TM domain by anchoring MGC to the plasma membrane controls almost all (more than 90%) the basal CCD activity. (v) Besides its core seven-CE elemental structure, the catalytic domain is embedded with two regulatory domains, NCδ and CO_2_; thus, CCD is both a core and regulatory catalytic center. (vi) CCD controls all four phototransduction pathways (discussed below).

### Photoreceptor ROS-GC1 Linkage with Phototransduction

Similar to its critical role in controlling the basic catalytic operation of the MGC family, CCD also plays a vital regulatory role in modulating all four ROS-GC1 interlocked phototransduction pathways. The mutation in any one of its seven CE elements severely impedes it’s all Ca^2+^-modulated rod and cone photoreceptor along with the Ca^2+^-modulated and CO_2_/bicarbonate-modulated catalytic activity. The cyclic GMP output in the cells is compromised, and it is predicted that finally the photoreceptors will die. Notably, the ligand-modulated EC_50_ and Hill coefficient values of the pathways are not affected, demonstrating that CEs core element has no control over the ligand binding activities of the ROS-GC.

### Model

The MGC signal transduction is a two-step process. Step one, it is contributed by the seven CE elements of the CCD and occurs in all members of the MGC family. The CCD captures Mg^2+^-GTP in its pocket and turns itself from the inactive basal to the active basal state (**Figure 3D**). Step two, it is a regulatory process. Upon ligand (GCAP1, GCAP2, S100B, or bicarbonate) binding, the rotation of each CCD monomer occurs around their two-fold axes (**Figure 3C**). This brings the CE residues into the closed maximally active position, and collectively their manifestation of the ligand dependent catalytic saturation activity.

## Conclusion

This study has predicted and experimentally solved the basic 3D-structure of the CCD existing in all members of the MGC family, demonstrated a unified code by which it operates; and, then applied this knowledge to explain some of the most fundamental principles by which the ROS-GC1 is interlocked with phototransduction in rods and cones. Finally and strikingly, the existing seven MGCs—ANF-RGC, CNP-RGC, STa-RGC, ROS-GC1, ROS-GC2, ONE-GC, GC-GC—in a specific fashion, by synthesizing cellular second messenger cyclic GMP, are linked with the physiological processes of blood pressure regulation, cellular growth, sensory transductions, neural plasticity, memory, temperature sensing (reviewed in Sharma et al., 2016) and, with tumor suppression (reviewed in Steinbrecher and Cohen, 2011; Windham and Tinsley, 2015). This study demonstrates that all these functions converge to a common site, CCD, which through a unified signal transduction mode, control these activities.

## Author Contributions

TD designed, carried out the experiments and analyzed their results. SR created and explained the stereo models. AP created and expressed all the mutants. RS conceptually planned and coordinated the study and sided in generating the model. All authors contributed to the writing of the manuscript.

## Conflict of Interest Statement

The authors declare that the research was conducted in the absence of any commercial or financial relationships that could be construed as a potential conflict of interest.

## Footnotes

1 http://zhanglab.ccmb.med.umich.edu/I-TASSER/

2 http://fatcat.sanfordburnham.org/
