# Supplementary material for: Membrane Guanylate Cyclase catalytic Subdomain: Structure and Linkage with Calcium Sensors and Bicarbonate
Source: Front Mol Neurosci. 2017 Jun 7;10:173. doi: 10.3389/fnmol.2017.00173 (PMC5461267; doi:10.3389/fnmol.2017.00173)
Supplement: Supplementary file 1 [file Presentation_1.PDF]

Iterative Threading and ASSEmbly Refinement, I-TASSER was used for modeling of the ROS-GC1 catalytic domain. I-TASSER is a protein modeling approach that involves the following three key steps:

A. Each simulation begins with the user input sequence (target). Secondary structure of the target sequence is predicted using machine learning approaches. The predicted secondary structure along with the input target sequence will then be fed as input to a group of threading programs (9 of them; more information is available from I-TASSER website), collectively called LOMETS (Local Meta-Threading-Server; http://zhanglab.ccmb.med.umich.edu/LOMETS/). LOMETS will then identify suitable templates for the target sequence based on the target-to-template Z-scores (normalized score; energy in standard deviation units relative to mean) from the PDB library (www.rcsb.org). Usually, one or two best Z-score templates from each threading programs will then be collected and used in the next step (see section B below). After identifying the templates, I-TASSER will split the templates into smaller continuous fragments.
B. Second step uses two different modeling techniques, replica-exchange Monte-Carlo simulation and ab initio modeling. Monte-Carlo is used for assembling the full-length models from the continuous PDB template fragments identified in step A. The unaligned regions are filled-in using ab-initio modeling approach. In this step, a software package called SPICKER (http://zhanglab.ccmb.med.umich.edu/SPICKER/) will be used to cluster the simulation models to identify low energy models.
C. In the final step, steric overlaps are removed by monitoring H-bonded networks to create a final model.

To calculate the atomic-level three dimensional structure of CCD default parameters of I-TASSER (webserver version) were used for the calculation.

Structural templates (experimental structures from PDB library) identified by LOMETS pipeline were used for building the final model(s) (please note that this information was provided in the original version of our manuscript: MATERIALS AND METHODS section; Molecular Modeling subsection; lines 6 and 7). The top 3 templates used for building models were 3uvj, 3et6 and 4p2f (experimental PDB IDs). Based on the protein family of these template proteins and the corresponding normalized Z-scores, we feel confident that our model represents the correct ROSGC1-catcd fold. Based on our analysis of comparing the model predictions with our experimental data (Results and Discussion sections of the manuscript), we strongly feel that our model is an improvement from the existing models. Please note that the Z-scores for the top 3 templates were 3.64, 3.45 and 3.85 respectively. To clarify, the Z-scores were derived from the threading alignments (LOMETS) and the ones that have scores greater than 1 are indicative of high-quality alignment models.

Restrained molecular dynamics simulation.

In this study, we were interested in deriving a highly reliable structural fold for the catalytic domain of ROS-GC1 and to predict the dimer conformation. We have taken the experimental information captured in 3et6 (PDB ID; Winger et al, BMC Struct.Biol. **8**: 42-42 2008) as a guidance for constructing the dimer form of CCD of ROS-GC1. More appropriate statistics in our case are the normalized B-factors and C-Scores. B-factor provides the inherent thermal mobility of the residues and in turn will indicate the stability of the protein. The normalized B-factor plot is shown below (taken from I-TASSER results):

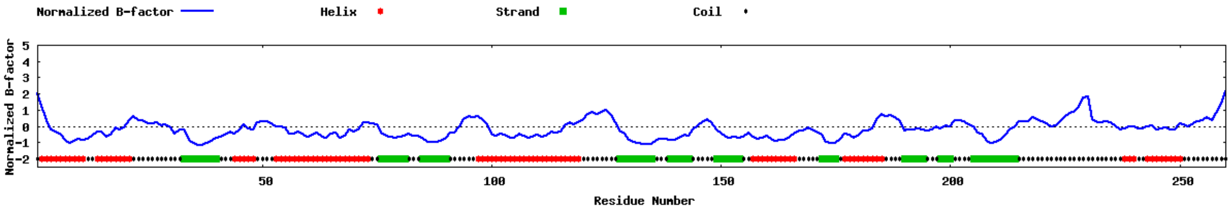

Each model also comes with confidence score called C-score [range -5,2]. Typically, higher value of C-scores indicate models with higher confidence. C-score for our top model was -1.85. Ramachandran plot for the top model (see below) shows that the number of residues in favored/allowed and outlier regions are 97.4% and 2.6% respectively and are within the expected range for a model structure. Ramachandran plot analysis was carried out using RAMPAGE (http://mordred.bioc.cam.ac.uk/~rapper/rampage.php) and the Ramachandran plot (see below) was created using Discovery Studio Visualizer (v.4.5.0; BIOVIA Inc).

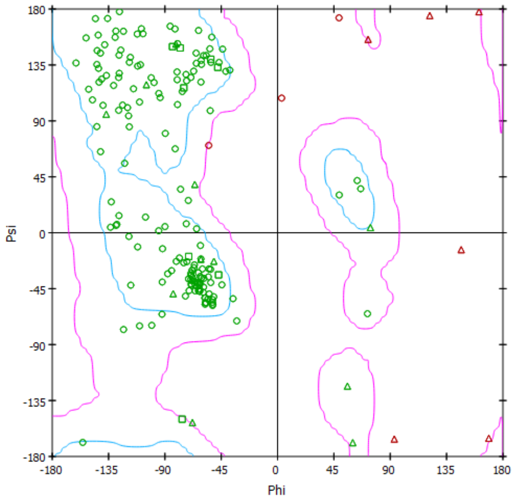

Another quantity that can add confidence to the model accuracy is the structural similarity of the model with the related analogs from the experimental PDB library. The table (see below) provides the RMSDs of the top 10 structural analogs.

Rank	PDB	RMSD
1	3uvjA	1.20
2	2wz1B	1.85
3	2w01A	2.56
4	4yusA	3.50
5	3r5gA	2.46
6	4p2fA	2.46
7	3et6B	2.09
8	1culA	2.47
9	1wc6C	2.51
10	4clfA	3.71

Comparison of the present model with the existing 1AWL model.

The 1AWL model (PNAS, 94, 13414, 1997), was constructed using one template (PDB 1AB8; rat type II adenylyl cyclase). Our model as described elaborately above was constructed using a newly developed mixed (threading + ab-initio; I-TASSER) approach and uses 10 templates identified by LOMETS (please refer to our reply for question #1). More importantly, I-TASSER was able to identify the newer experimental eukaryotic GC structure (PDB: 3et6; Winger et al (2008)) template without any human intervention. To the question of how is our new model different from the previous model, please note that our model covers significantly longer region than 1AWL by about 30 residues in the C-terminal end, our model captures the experimental fold as described by PDB ID, 3et6. To visualize the differences we overlapped the 1AWL and our models (shown in the same orientation as our manuscript Fig 3A; our model: Cyan color and 1AWL: Pink color). Significant differences are obvious in the following key secondary elements: loop α1-α2, α3 and β4a, β5 and β4b, a5 and β7, etc. The protein structures were aligned using FATCAT online server (http://fatcat.burnham.org/fatcat) using rigid alignment model with default parameters.

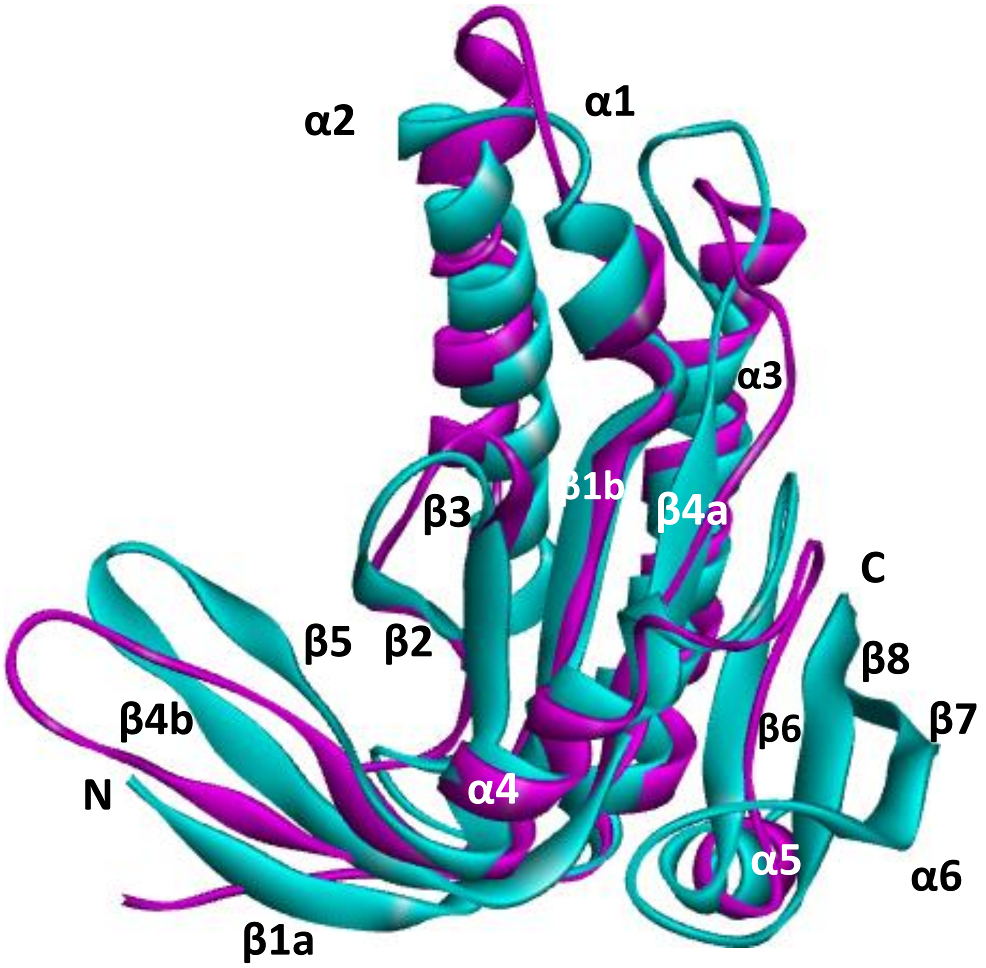

1AWL: Pink
ROSGC1-catcd model: Cyan
